# Superoscillation: from physics to optical applications

**DOI:** 10.1038/s41377-019-0163-9

**Published:** 2019-06-12

**Authors:** Gang Chen, Zhong-Quan Wen, Cheng-Wei Qiu

**Affiliations:** 1 College of Optoelectronic Engineering, Chongqing University, 174 Shazheng Street, Chongqing, 400044 China; 2 Department of Electrical and Computer Engineering, National University of Singapore, 4 Engineering Drive 3, Singapore, 117583 Singapore

**Keywords:** Sub-wavelength optics, Nanophotonics and plasmonics

## Abstract

The resolution of conventional optical elements and systems has long been perceived to satisfy the classic Rayleigh criterion. Paramount efforts have been made to develop different types of superresolution techniques to achieve optical resolution down to several nanometres, such as by using evanescent waves, fluorescence labelling, and postprocessing. Superresolution imaging techniques, which are noncontact, far field and label free, are highly desirable but challenging to implement. The concept of superoscillation offers an alternative route to optical superresolution and enables the engineering of focal spots and point-spread functions of arbitrarily small size without theoretical limitations. This paper reviews recent developments in optical superoscillation technologies, design approaches, methods of characterizing superoscillatory optical fields, and applications in noncontact, far-field and label-free superresolution microscopy. This work may promote the wider adoption and application of optical superresolution across different wave types and application domains.

## Using superoscillations in light for superresolution imagery

Researchers are getting closer to achieving ‘superresolution’ images by employing localized high-amplitude oscillations in light waves. Cheng-Wei Qiu of the National University of Singapore and colleagues in China reviewed developments in optical ‘superoscillation’ technologies, which aim to overcome current limitations in superresolution techniques requiring contact with the observed object, the use of fluorescent labels, or viewing that is restricted to the near-field of a lens. Superoscillation is a mathematical phenomenon in which a light wave contains local frequencies that are large in amplitude. Optical systems employing this phenomenon could improve the ability to distinguish two tiny objects separated by nanoscale-length distances. Recent developments show potential for applications in telescopes, microscopy, and ultrahigh density optical data storage. Improving the design of superoscillatory lenses could overcome challenges in efficiently focusing more of the incident optical energy.

## Introduction

Due to the propagation property of electromagnetic waves, the optical resolution of conventional optical systems is restricted to a basic theoretical limit of 0.61λ/NA (NA is the numerical aperture of the optical system)^1^. For optical waves with a wavelength of λ, in a homogeneous lossless medium with refractive index *n*, the propagation of light acts as a band-limited linear space-invariant system^2^ that filters out all components for which the spatial frequency exceeds *n*/λ within a distance of several wavelengths. The absence of higher frequency components results in limited optical resolution. To overcome this restriction, retrieving higher frequency components from evanescent waves, which exist near the objective surface within a distance of less than one wavelength, is necessary. Near-field optical-scanning microscopes^3^ exploit this property to achieve subdiffraction resolution of tens of nanometres using a nano-optical probe. Dielectric microspheres on sample surfaces can also yield subdiffraction features of the sample in the form of magnified virtual images, which can then be captured with a conventional optical microscope^4,5^, by relying on the conversion of evanescent waves into propagation waves. Superresolution imaging based on metallic and dielectric superlenses^6–8^ has been demonstrated in the near-field regime. Optical hyperlenses were also developed for far-field imaging beyond the diffraction limit by using anisotropic metamaterials^9–11^, which can convert evanescent waves into propagating waves with very large wavenumbers. High-frequency components in evanescent waves can also be attained in the far field via spatial frequency shifting. According to the angular spectrum theory, using spatially modulated light, the high-frequency components in evanescent waves can be shifted to low-frequency components and then converted to propagating waves. In this way, the high-spatial-frequency information can be retrieved in the far field for the reconstruction of superresolution images, which has been demonstrated by a variety of structured light illumination microscopes (SLIMs) with complicated postprocessing^12^. The resolution of a conventional SLIM is twice that of a traditional optical microscope with the same NA. Higher resolutions have been reported for structured light using surface plasmonic waves^13^ and nanowire fluorescence^14^. However, such illumination is restricted to within the near-field region of several tens of nanometres on the surfaces of nanowires, which prohibits deep imaging inside a sample. Nonlinear optics is also applied to enhance the superresolution via saturated structured fluorescence illumination^15^. Stimulated emission depletion (STED) employs two laser pulses for excitation and de-excitation of fluorophores to achieve superresolution through the nonlinear dependence of the simulated emission rate on the intensity of the de-excitation beam^16^.

In addition to purely optical techniques, by utilizing sequential activation and time-resolved localization of photoswitchable fluorophores, superresolution images can be reconstructed using stochastic techniques, including stochastic optical reconstruction microscopy^17^, photoactivated localization microscopy^18^ and fluorescence photoactivation localization microscopy^19^. Utilizing surface-enhanced Raman scattering, label-free superresolution microscopy was also demonstrated by a stochastic method^20^. Utilizing deep-learning approaches, the resolution can be further improved for bright-field microscopic imaging^21^ and fluorescence microscopy^22,23^. These methods, however, require either fluorescence labelling or postprocessing to achieve superresolution. In this context, far-field label-free direct superresolution imaging, without close contact with samples, is favourable for many applications, such as optical microscopy, telescopy and data storage. Recently, the concept of superoscillation has been tailored to and applied in optical superresolution both theoretically and experimentally to provide an alternative way to achieve label-free noncontact optical superresolution in the far field.

## Optical superoscillation

Superoscillation refers to the phenomenon in which a band-limited function can contain local oscillations that are faster than those of the fastest Fourier components. Superoscillation allows the formation of arbitrarily small optical features, which can be used for superresolution focusing and imaging. Before proposing the concept of optical superoscillation, substantial endeavours were made in realizing resolution beyond the diffraction limit, such as apodization^24–26^ and the use of pupil filters^27–32^. Mathematically, if a two-dimensional (2D) analytic function is known exactly in an arbitrarily small spectral region, then the entire function can be determined uniquely by means of analytic continuation^33^. The diffraction limit of an optical imaging system can be overcome to some extent at the expense of the system performance in other areas. An optical system can theoretically attain as high a resolution capability as desired^34^. For an object of limited size, arbitrarily perfect imaging can be obtained under various conditions by coating the lens to realize a particular transmission function at the expense of tremendous loss of illumination, which also leads to a severely narrow field of view (FOV)^35^. A similar strategy has been suggested that utilizes superdirective antennas^36^, and it was investigated in the optical domain to improve the optical resolution capability. An arbitrary resolving power can be achieved by applying properly designed concentric ring pupils.

The concept of superoscillation was originally defined in terms of the quantum weak measurements by Aharonov^37^ and was later developed and extended to optics by Michael Berry^38–47^, who suggested the possibility of demonstrating optical superoscillation without evanescent waves via subwavelength grating diffraction^39^. Using a paraxial approximation, the propagation of superoscillations was solved for subwavelength gratings, thereby revealing a direct connection with the Talbot effect in diffraction theory. This theory also predicts the formation of superoscillatory fine structures with spatial features of wavelength λ/4, which were experimentally observed in the diffraction pattern of superfocusing due to the nonlinear Talbot effect^48^.

**Fig. 1:**
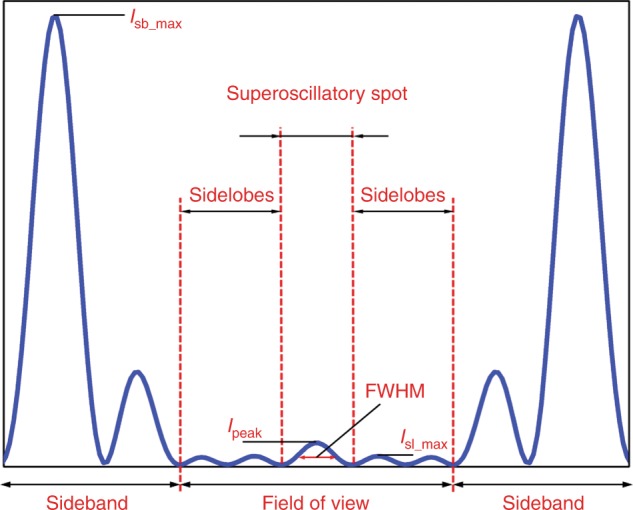
In-plane intensity distribution of a superoscillatory optical spot. The entire plane can be divided into the area of the FOV and the area of the sideband. Within the FOV, the superoscillatory spot is surrounded by sidelobes with intensities that are smaller than the spot peak intensity. Outside the FOV, there are sidebands with intensities that well exceed the spot peak intensity. *I*_peak_, *I*_sl_max_ and *I*_sb_max_ are the intensities of the superoscillatory spot, the maximum sidelobe and the maximum sideband, respectively, and FWHM is the full width at half maximum of the spot

For optical waves, superoscillations correspond to local spatial frequencies (the gradient of the phase distribution) which exceed the wavenumber, and they are associated with phase singularities^40^. Researchers have observed the superoscillatory nature of the band-limited complete set of prolate spheroidal wavefunctions (PSWFs) *φ*_n_(c,r)^49^, which have *n* zeros within the finite area of [−*c*/*k*, *c*/*k*] (*k* is the wavenumber) and enable the synthesis of arbitrarily small features by linear superposition^50^. However, with the increase in the number of superoscillatory features or the increase in the number of zeros *n*, the superoscillation area [−*c*/*k*, *c*/*k*] exhibits a dramatic reduction in its confined energy, while the energy that is contained in the sideband outside of [−*c*/*k*, *c*/*k*] increases tremendously, which requires a significant increase in the total energy to generate such superoscillatory features^51^. Figure 1 depicts an optical superoscillatory distribution, which can be divided into two areas: the FOV and sideband areas. The corresponding electric field can be characterized by five parameters: the spot full width at half maximum (FWHM), the peak intensity (*I*_peak_), the sidelobe ratio (the ratio of the maximum sidelobe intensity to the peak intensity within the FOV, namely, *I*_sl_max_/*I*_peak_), the FOV and sideband ratio (the ratio of the maximum sideband intensity to the peak intensity outside of the FOV, namely, *I*_sb_max_/*I*_peak_). Imaging applications require reduction of the spot size, increase of the spot intensity and reduction of the sidelobe ratio, while extending the FOV and suppressing the sideband ratio. However, in most cases, tradeoffs must be made among the five parameters, especially when the spot size is much smaller than the diffraction limit.

Another characteristic of the optical superoscillatory field is the sharp phase change at the zero amplitudes. The phase distribution plays a role in the generation of optical superoscillatory features^52,53^. The optical field can be described by its electric field $E( {\mathop{r}\limits^{\rightharpoonup} }) = A( {\mathop{r}\limits^{\rightharpoonup} })\exp [ {i\varphi( {\mathop{r}\limits^{\rightharpoonup} })} ]$, where $A( {\mathop{r}\limits^{\rightharpoonup} })$and$\varphi( {\mathop{r}\limits^{\rightharpoonup} })$denote the amplitude and phase, respectively. By substituting the above expression into the Helmholtz equation, one obtains the following equations:

$$\left\{ {\begin{array}{*{20}{l}}{{\nabla ^2}\varphi \left( {\mathop{r}\limits^{\rightharpoonup} } \right) + \nabla \left( {\ln \,{A^2}\left( {\mathop{r}\limits^{\rightharpoonup} } \right)} \right) \cdot \nabla \varphi \left( {\mathop{r}\limits^{\rightharpoonup} } \right) = 0} \\{{\nabla ^2}A\left( {\mathop{r}\limits^{\rightharpoonup} } \right) + \left[ {{k^2} - {{\left| {\nabla \varphi \left( {\mathop{r}\limits^{\rightharpoonup} } \right)} \right|}^2}} \right]A\left( {\mathop{r}\limits^{\rightharpoonup} } \right) = 0}\end{array},\,{\text{where}}\,{k}\,{\text{is}}\,{\text{the}}\,{\text{wavenumber}}{.}} \right.$$

**Fig. 2:**
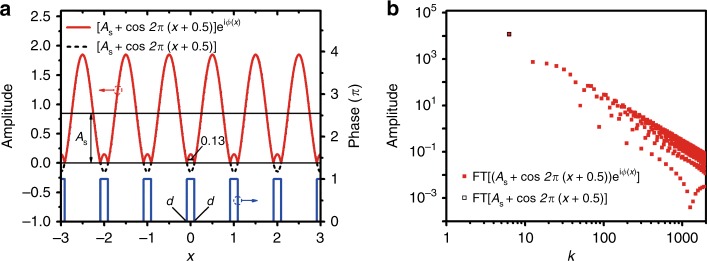
Example of a reduction in frequency of the entire superoscillatory field with the proper phase distribution. **a** The amplitude of the 1D optical field is given by *f*(*x*) = {[*A*_*s*_ + cos[2π(*x* *+* 0.5)]}·exp[i*φ*(x)], where the corresponding phase is given by *φ*(*x*) = π × ∑Rect(2π*n* + *x*/2*d*) and rect(∙) is defined as 1 within [−0.5, 0.5] and 0 elsewhere, and **b** a comparison of the Fourier spectra between the optical field amplitude *f*(*x*) and the entire optical field *E*(*x*) = *f*(*x*)∙exp[−*iφ*(x)] = *A*_*s*_ + cos[2π(*x* *+* 0.5)], consisting of components with wavenumber values of 0 and 1, which are much smaller than those of the amplitude

The gradient of the phase distribution, $\nabla \varphi \left( {\mathop{r}\limits^{\rightharpoonup} } \right)$, yields the local wavenumber. When the length of the local wavenumber, namely, $\left| {\nabla \varphi (\mathop{r}\limits^{\rightharpoonup} )} \right|$, well exceeds the wavenumber *k* at point $\mathop{r}\limits^{\rightharpoonup}$, the second equation implies a fast decay in the electrical amplitude$A\left( {\mathop{r}\limits^{\rightharpoonup} } \right)$ in the neighbouring area that surrounds this point, which leads to the formation of superoscillatory structures. A numerical study also demonstrated that the special phase distribution is crucial in reducing the Fourier frequency of the entire electric field and keeps the Fourier components within the range that is limited by the wavenumber *k* ^53^. An example of a one-dimensional (1D) optical field is presented in Fig. 2, in which the phase discontinuity at *x* = 2π*n* ± *d* results in local wavenumbers with infinite values.

Moreover, the phase distribution directly determines the backflow phenomenon in the optical superoscillation features^54^, in which optical waves with only positive momenta can travel backward with a negative local wavenumber. The backflow is closely related to superoscillation^39,55,56^ and was recently demonstrated experimentally in 2D superoscillatory optical fields^57^; in addition, the four major characteristic features of a superoscillatory optical field were identified: a highly localized field, phase singularities, extremely large local wavevectors and energy backflow.

**Fig. 3:**
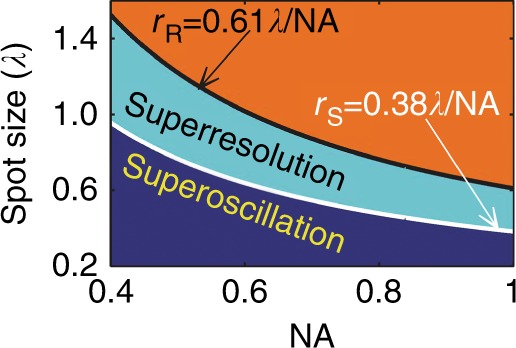
Focal spot sizes for different values of the NA, which equals the sine (sinα) of the angle between the optical axis and the maximum convergent ray in free space. The two curves, which correspond to the Rayleigh (black) and superoscillation (white) criteria, divide the focal spots into three types: subresolved (orange), superresolved (cyan) and superoscillation (dark blue). Reproduced with permission from ref. ^58^ Copyright 2014, John Wiley and Sons

For a focusing lens with NA = *n*sin*α*, the size of the circularly symmetrical optical spot is determined by the highest spatial frequency *k*_*r*_ = *k*sin*α*, where *n* is the refractive index of the medium after the lens and *α* is the angle between the optical axis and the wavevector. The spot size corresponds to the distance between the central peak and the first zero of the zero-order Bessel function of the first kind, which yields a value of 0.38λ/NA and is close to the FWHM in most cases. According to this analysis, Qiu^58^ suggested 0.38λ/NA as the criterion for optical superoscillation focusing. As illustrated in Fig. 3, the graph of spot size vs. NA is divided into three areas: the area with a spot size that exceeds the Rayleigh diffraction limit is defined as the subresolved area, the area with a spot size smaller than 0.38λ/NA is defined as the superoscillation area, and the area in between is defined as the superresolution area.

Although further theoretical and experimental efforts are still required to fully understand the physics behind optical superoscillation, it has already proven to be a wonderful tool for engineering far-field superresolution optical elements and optical systems^59^.

## Superoscillatory optical devices

### Focusing linearly and circularly polarized waves

**Fig. 4:**
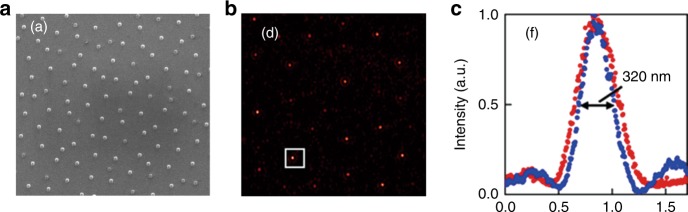
First experimental observation of optical superoscillation. **a** An SEM image of a quasiperiodic metallic nanohole array, and **b** its corresponding diffraction pattern at 7.5λ from the array. **c** The optical intensity distribution of the superoscillatory spot (marked by the square) along vertical (red) and horizontal (blue) directions in **b**. Reproduced with permission from ref. ^60^ Copyright 2007, AIP Publishing

Optical superoscillation was first observed in the diffraction pattern of a quasiperiodic metallic nanohole array under the illumination of linearly polarized monochromatic light at a wavelength of 660 nm^60^. In the experiments, hot spots with an FWHM of 0.36λ were generated in the absence of evanescent waves at a far-field distance of 7.5λ, as shown in Fig. 4. This type of binary amplitude (BA) mask provides a promising method for the experimental realization of superoscillation optics.

Most of the early studies on superoscillation optics focused on the subdiffraction focusing of monochromatic light in the visible range. Because of their robustness and ease of fabrication, most of the reported superoscillation focusing lenses are based on BA ring masks that are fabricated in thin metal films, such as germanium, gold and aluminium, which are deposited on glass substrates. Such devices consist of multiple concentric rings with an amplitude transmission of either 0 or 1. The judicious design of the transmission pattern aims at forming hot spots with an FWHM that is less than the traditional diffraction limit of 0.5λ/NA through constructive and deconstructive interference on the focal plane. Using multiple concentric air nanorings with a diameter of 8.46λ (4.5 μm), subdiffraction focusing was demonstrated with an FWHM of 0.6λ at *z* = 5.26λ (2.8 μm)^61^, which is slightly smaller than the corresponding Abbe diffraction limit of 0.63λ (0.5λ/NA), and the maximum sidelobe intensity was more than 40% of the intensity of the central peak. A standard superoscillatory lens (SOL) based on a BA ring mask was optimized and designed with a focal length of 16.1λ and a radius of 62.5λ (40 μm) at a wavelength of 640 nm. A hot spot was generated with an FWHM of 0.289λ (185 nm); however, the superoscillation focal spot was surrounded by a large sidelobe with almost the same intensity as the spot, which limited the FOV to ~0.6λ^62^. Multiple subdiffraction foci were also numerically demonstrated in an oil immersion medium with a refractive index of 1.515 for linearly polarized lights^63^. Although subdiffraction focusing was realized in the above cases, it only reflected the focal spot size of the transversely polarized light. Due to the polarization selectivity, which will be discussed later, the optical intensity obtained through a conventional optical microscope^64^ contains no information on the longitudinal components, which might broaden the focal spot size of the entire optical field.

According to ref. ^53^, increasing the modulation freedom in terms of the phase and amplitude (namely, increasing the number of phase and amplitude values used in the mask) can improve the superoscillation focusing performance, e.g., it can enhance the efficiency, reduce the sidelobes and extend the FOV. To increase the efficiency and suppress the large sidelobes near the superoscillatory focal spot without significantly increasing the fabrication difficulties, an additional binary phase (BP, 0 and π) modulation can be introduced into the mask design. Considering the contributions from longitudinal components, a superoscillation focusing lens based on a binary amplitude and phase (BAP) mask was proposed with an ultralong focal length of 400λ and an NA of 0.78 for circularly polarized light. A tilted nanofibre probe was employed to obtain the optical intensity distribution on the focal plane. The measured focal spot had an average FWHM of 0.454λ^65^, which is smaller than the superoscillation criterion of 0.487λ (0.38λ/NA)^58^. Clearly suppressed sidelobes were observed with an intensity less than 26% of the focal spot intensity. Other designs of SOLs that utilize BP^66^ and BAP^67^ masks were also reported for circularly polarized light.

**Fig. 5:**
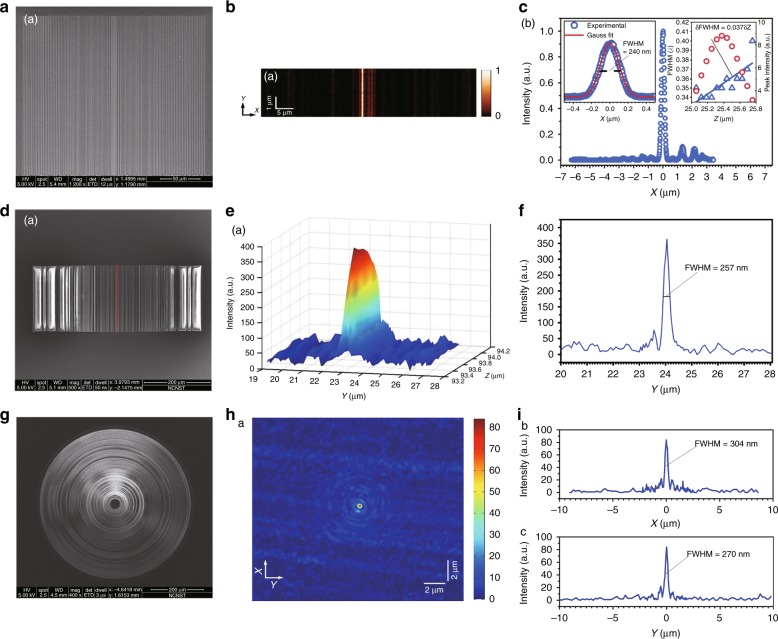
Superoscillatory lenses for linear-focusing and point-focusing. Superoscillatory optical lenses based on **a** metallic slits, **d** metallic and dielectric strips and **g** metallic and dielectric concentric rings for linear polarized light and circularly polarized light; **b**, **e** and **h** present the corresponding 2D intensity distributions; and **c**, **f** and **i** present the intensity distribution curves in the *x*- and *y*-directions for lenses **a**, **d** and **g**, respectively. **a**–**c** Reproduced with permission from ref. ^68^ Copyright 2016, IEEE. **d**–**f** Reproduced with permission from ref. ^69^ Copyright 2016, OSA. **g**–**i** Reproduced with permission from ref. ^65^, Copyright 2016, NPG, under a Creative Commons license (https://creativecommons.org/licenses/by/4.0/)

In addition to the point-focusing lens, linear-focusing SOLs have been demonstrated theoretically^53^and experimentally^68,69^. Linear-focusing SOLs are realized with metallic and dielectric strip arrays on top of glass substrates for amplitude and phase manipulation. When illuminated with TE waves, the diffraction pattern consists of only transverse polarization components; therefore, achieving superoscillation focusing with small sidelobes is much easier due to the absence of longitudinal components. Both a quasicontinuous amplitude mask^68^ and a BAP mask^69^ were applied to linear-focusing SOLs. Quasicontinuous amplitude modulation was realized by varying the width of the subwavelength metallic slit. According to the acquired total optical intensity, a focal line FWHM of 0.379λ (larger than the theoretical prediction of 0.34λ but slightly smaller than the superoscillation criterion of 0.39λ) was experimentally demonstrated, with a small sidelobe ratio of 10.6%^68^. Figure 5 presents the experimental results of superoscillatory optical lenses based on metallic slits^68^, metallic and dielectric strips^69^ and metallic and dielectric concentric rings^65^.

SOLs based on metallic and dielectric concentric rings or strip arrays might suffer from polarization selectivity when the size of the rings is much smaller than half the wavelength. Polarization-independent subwavelength wave-front manipulation structures are favourable for many applications in which incident waves have complex polarization distributions. Subwavelength structures with circular symmetry are insensitive to polarization. A periodic double-layer metallic hole array^70^ has been proposed for the continuous modulation of phase and amplitude to realize an SOL with a theoretically predicted focal spot FWHM of 0.319λ and a sidelobe ratio of 30%. Accurate control of the amplitude and phase can also be achieved with polarization-independent aperiodic photon sieves^71^, in which subwavelength holes are arranged in a nonperiodic concentric fashion. By optimizing the radius of the rings (10.85–19.65 μm) and the diameter of the holes (50–100 nm), for linearly polarized light, a sub-diffraction-limit focal spot was demonstrated experimentally with an FWHM of 0.316λ in the transverse polarized optical field at 21λ from the lens in air^71^. The size of the spot is much smaller than the superoscillation criterion of 0.458λ (0.38λ/NA); however, the central peak is surrounded by a large sidelobe, as previously reported^62^.

### Focusing cylindrically polarized vector waves

Cylindrically polarized vector waves are linear superpositions of electric field components oriented in the radial and azimuthal directions. Because of their special polarization orientation and tight focusing ability^72,73^, they are important in a variety of applications, including particle manipulation^74,75^, superresolution optical microscopy^76,77^, lithography^78^, material processing^79^ and particle acceleration^80^.

Traditionally, a tight focus with longitudinal polarization can be realized by focusing radially polarized waves with a high–NA lens in combination with an annular aperture filter^81^. Based on constructive interference, a lens consisting of subwavelength concentric annular metallic grooves was proposed for subdiffraction focusing of waves with radial polarization (RP) by scattering the surface plasmon polaritons (SPPs). A hot spot with an FWHM of 0.40λ was theoretically predicted at several wavelengths from the groove surface^82^. Utilizing a similar mechanism, a far-field plasmonic lens was experimentally demonstrated that can focus dual-wavelength (λ = 632.8 nm and 750 nm) waves to the same focal plane and the obtained FWHM of the focal spots was 0.41λ for both wavelengths on the focal plane that is located at a distance of 1.2 μm^83^. Using the polarization selectivity of SPPs^84^, an SPP lens was designed that generates and focuses in-phase radially polarized light under the illumination of waves with linear polarization (LP)^85^. Constructive interference of the scattered SPP waves was guaranteed in the far field 20 μm from the lens surface by tuning the propagation constant of the SPPs via the slit width. The experimentally obtained FWHMs were 340 ± 60 nm (0.38λ/NA) and 420 ± 60 nm (0.47λ/NA) in the *x*- and *y*-directions, respectively^85^, in water. However, the intrinsic loss that is caused by SPP propagation might significantly reduce the efficiency of the SP lens. To avoid SPP loss, an SOL based on a BA^86,87^ and BP^66^ annular ring belt array can be designed for the superoscillation focusing of radially polarized waves. A BP SOL fabricated with a Si_3_N_4_ concentric ring array that exhibits a subdiffraction longitudinally polarized focal spot with an average FWHM of 0.456λ and a depth of focus (DOF) of 5λ has been demonstrated^88^. Sharper focal spots were expected using higher-order radially polarized waves^89,90^. In addition to longitudinal electric fields, the creation of a purely longitudinal subdiffraction magnetic field was studied numerically in optomagnetic materials under the inverse Faraday effect by tightly focusing an azimuthally polarized vortex beam^91^.

**Fig. 6:**
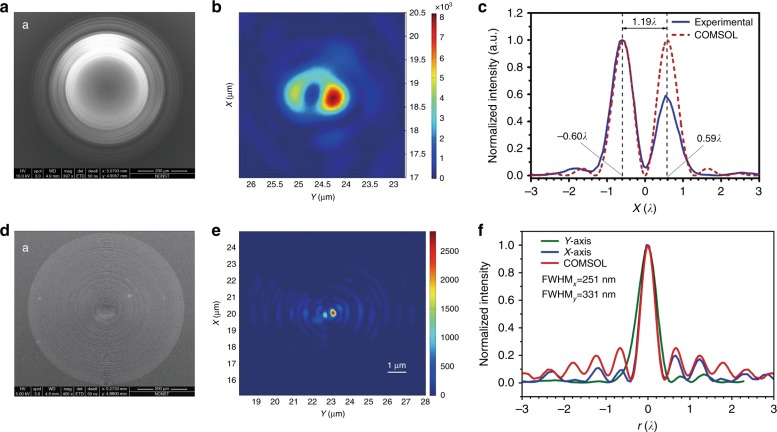
Superoscillatory lenses for cylindrically polarized waves. Superoscillatory optical lenses for focusing **a** azimuthally and **d** radially polarized light. **b** and **e** are the 2D optical intensity distributions of the diffraction patterns on the focal planes of lenses **a** and **d**, respectively; **c** and **f** are the corresponding intensity distribution curves extracted from **b** and **e** in the *x*- and *y*-directions. Reproduced with permission from ref. ^88,95^. **a**–**c** Ref. ^95^ Copyright 2016, NPG, **d**–**f** ref. ^88^ Copyright 2016, NPG, under a Creative Commons license (https://creativecommons.org/licenses/by/4.0/)

The generation of subdiffraction 2D hollow spots is critical to STED microscopy^92^ and nonlinear nanolithography^93^. Reducing the inner radius of the 2D hollow spot is of particular importance in further enhancing the spatial resolution. Traditionally, 2D hollow spots can be created by focusing a helical-phase optical vortex beam with a high-NA objective lens. An alternative way to generate a tight 2D hollow spot is to focus azimuthally polarized waves^94^. Subdiffraction focusing of azimuthally polarized waves was demonstrated with SOLs that were based on BP dielectric Si_3_N_4_^95^ and BA metallic aluminium^96^ concentric ring arrays. The inner FWHMs of the generated 2D hollow spots were 0.61λ (0.39λ/NA) and 0.368λ (0.352λ/NA), respectively, which are slightly larger than their theoretically predicted values of 0.57λ and 0.349λ. Figure 6 shows the experimental results of focusing cylindrically polarized vector waves.

Most of the reported SOLs for focusing cylindrically polarized vector waves are based on either a BA or BP concentric ring array. Although the fabrication is comparatively easy, these arrays offer very few degrees of freedom in the optimization of the wave-front modulation mask. Recent rapid developments in metasurfaces have enabled flexible control of the amplitude^97^, phase^98–101^ and polarization^102,103^ on a subwavelength scale. A group of eight metallic antennas was proposed for the modulation of cross-polarized transmission light, which cover a phase range of 2π. Based on such antennas, metalenses were designed with the integrated functions of polarization conversion and focusing. The incident azimuthally polarized waves can be converted into radially polarized waves and focused into a longitudinally polarized solid spot, while incident radially polarized waves can be converted into azimuthally polarized waves and focused into a 2D hollow spot. A numerical simulation demonstrated a longitudinally polarized focus with a subdiffraction size of 0.47λ and an azimuthally polarized hollow focus with a subdiffraction size of 0.43λ^104^.

In focusing cylindrically polarized vector waves, aligning the optical focusing device to the optical axis of the incident waves is difficult, especially in the case of subwavelength and subdiffraction focusing. Any misalignment can result in deformation of the focal spot and even destruction of the subdiffraction features. The best solution is to integrate the polarization conversion and focusing functions into a single device for incident waves with either circular polarization or LP. Under the illumination of circularly polarized waves, the orientation of the linearly polarized reflection wave can be continuously tuned by rotating a reflective quarter-wave plate (QWP) metasurface. In combination with BA (0 and π) modulation, a subdiffraction longitudinally polarized focus was numerically demonstrated at a wavelength of 1064 nm with a metamirror consisting of five concentric rings^105^. A family of cross-shaped reflective QWP metasurfaces was proposed with 32 equally separated phase values over the 2π range at a wavelength of 1550 nm. Two subdiffraction focusing mirrors based on the QWP metasurfaces were demonstrated over a broad bandwidth of 210 nm, which converted circularly polarized incident waves into radially polarized and azimuthally polarized waves and focused them into longitudinally polarized spots with FWHMs of 0.38λ‒0.42λ and azimuthally polarized 2D hollow spots with inner FWHMs of 0.32λ‒0.34λ^106^. In the design of a QWP-based focusing metasurface, it is necessary to compensate for the additional geometrical phase that is induced by the rotation by applying elements that are selected according to the polar angles, which might result in a slightly nonsymmetric intensity distribution in the focal spot. This problem can be overcome by utilizing half-wave plate (HWP) metasurfaces. A subdiffraction focusing lens based on HWP metasurfaces has been demonstrated with a group of dielectric elliptical cylinder metasurfaces of 30 sizes at a wavelength of 915 nm. The dielectric metalens converts linearly polarized waves into radially polarized waves, which are focused into longitudinally polarized subdiffraction spots with FWHMs of 0.385λ‒0.458λ in a broad wavelength range of 875‒1025 nm^107^.

### Subdiffraction optical needles and diffractionless beams

The conventional elements for realizing an extended DOF include axicons^108^, diffraction gratings^109,110^, aberration lenses^111^, and Fresnel zone plates^112^. Subdiffraction optical needles are focal spots that extend along the optical axis with a transverse size that is smaller than the Abbe diffraction limit. Such optical needles are ideal for particle acceleration, superresolution imaging, high-density data storage and fabrication of planar structures.

A linearly polarized superoscillatory optical needle was first demonstrated at a wavelength of 640 nm with a lens that has been modified from a conventional point-focusing BA SOL by simply blocking its central area with a circular metallic disk^113^. The optical needle had an axial length of 11λ and a transverse size of 0.42λ in the experiment. An optimization method was also employed to design optical needle SOLs. At a violet wavelength of 405 nm, a BA SOL was optimized, and the generation of a circularly polarized optical needle with a long DOF of 15λ and a transverse size of 0.45λ was experimentally demonstrated^114^. A theoretical design of BP lenses for generating deep ultraviolet optical needles was also reported^115^. However, these reported lenses have very short focal lengths of approximately 20‒30λ, which poses a major obstacle to practical applications. To overcome this problem, an SOL with an ultralong focal length of 240λ was developed for focusing an azimuthally polarized vortex beam with a topological charge of *m* = 1. The transverse size of the generated optical needle varied between 0.42λ and 0.49λ within the 12λ propagation distance^116^. Using a BP mask, a subdiffraction optical needle was experimentally generated with a length of 19.7λ at a THz wavelength of 118.8 μm^117^.

The generation of a subdiffraction needle of a longitudinally polarized wave by focusing a radially polarized Bessel–Gaussian beam using a combination of a BA filter and a high-NA objective lens was theoretically proposed. The predicted propagation distance is approximately 4λ and the transverse size of the needle is 0.43λ^118^. A further theoretical study showed that the transverse size can be further reduced to 0.4λ within a DOF of 4λ with the proper beam intensity profile^119^. Using an SPP lens, a longitudinally polarized optical needle with a transverse size of 0.44λ and a length of 2.65λ was demonstrated at a meso-field distance by numerical simulation^120^. With an optimized SOL based on a BP mask, a 5λ-long longitudinally polarized optical needle was experimentally obtained with a transverse FWHM of 0.456λ (0.424λ/NA) at an ultralong working distance of 200λ^88^.

For superoscillation focusing fields, the sidelobe intensity typically increases substantially as the beam size is reduced below the superoscillation criterion of 0.38λ/NA. To suppress the sidelobes, a supercritical lens was proposed for realizing a transverse FWHM that is less than the diffraction limit but slightly exceeds the superoscillation criterion^121^. A 12λ-long optical needle was experimentally generated 135λ from the lens with a transverse size of 0.407λ and suppressed sidelobe intensity, which was only 16.2% of the central peak intensity.

In addition to subdiffraction solid optical needles, their hollow counterparts, which have zero intensity along the optical axis, are attractive for superresolution applications. In STED microscopy, improvements in the imaging depth with a hollow Bessel beam have been verified^122^. A hollow optical needle with a length of 2.28λ and a transverse inner size of 0.6λ, which was obtained by shaping a radially polarized Bessel-Gaussian beam with a second-order vortex phase and amplitude filter, has been theoretically reported^123^. Assisted by an optimization algorithm, a BP SOL was designed with a working distance of 300λ (189.84 μm), and a subdiffraction hollow needle was experimentally created with a length of 10λ by focusing azimuthally polarized waves. Within the needle, the transverse inner size varied between 0.34λ and 0.52λ, which is <0.5λ/NA^124^.

**Fig. 7:**
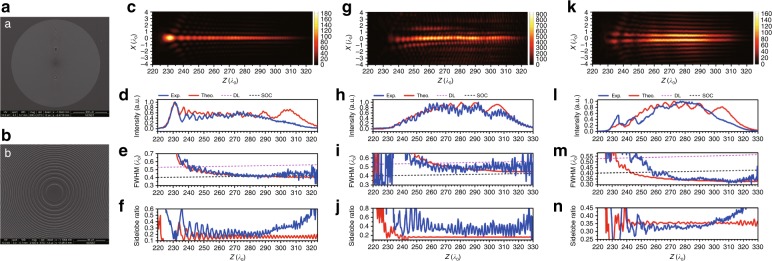
Generation of subdiffraction diffractionless beams. **a** An SEM image of a lens for the generation of subdiffraction diffractionless beams with different polarizations, including circular, longitudinal and azimuthal polarizations. **b** is a magnified image of **a**; **c**, **g** and **k** are 2D intensity distributions in the propagation plane for circularly, longitudinally and azimuthally polarized subdiffraction diffractionless beams; **d**–**f**, **h**–**j** and **l**–**n** present the theoretical and experimental results of the intensity, FWHM and sidelobe ratio along the optical axis for the three cases. Reproduced with permission from ref. ^130^ Copyright 2018, The Optical Society of America

The length of the optical needle can be further extended, but the required computational load renders the design of optical needles with lengths of hundreds of wavelengths challenging. Theoretical efforts have been made^125–128^, but there has been no experimental demonstration of such long subdiffraction optical needles. Recently, the concept of angular spectrum compression was proposed for generating ultralong subdiffraction optical needles, which significantly reduces the design complexity. Based on this concept, a superoscillation point-focusing lens that uses a BP mask was optimized at a wavelength of 672.8 nm. When illuminated with an azimuthally polarized wave at a shorter wavelength of 632.8 nm, the lens generated an optical hollow needle with a subdiffraction and subwavelength transverse size within the nondiffracting propagation distance of 94λ^129^. A numerical simulation also revealed that when the lens was immersed in water, the propagation distance was further extended to 180λ, while the beam remained superoscillatory with a transverse size of ~0.35λ–0.4λ. This result demonstrates the satisfactory penetration ability of the superoscillatory hollow needle, which is crucial for practical applications. In a later study, surprisingly, classical binary Fresnel zone plates were used to generate subdiffraction optical needles for multiple polarizations via optimization-free design^130^. A numerical simulation showed that, compared to the optical needle reported in reference^129^, those that were created by classical Fresnel zone plates have smaller fluctuations in optical intensity along the optical axis. The experimental results showed that the transverse sizes and the axial lengths were 0.40λ–0.54λ and 90λ, 0.43λ–0.54λ and 73λ and 0.34λ–0.41λ and 80λ for the generated optical needles with circular, longitudinal and azimuthal polarizations, respectively, as shown in Fig. 7. The realization of a longer needle is possible by further increasing the radius of the binary Fresnel zone plate or using a shorter working wavelength. Compared with binary concentric ring arrays, spatial light modulators (SLMs) can provide more opportunities in the design of lens phase profiles and, therefore, can be used to realize optical needles with complicated features. Diffractionless beams with arbitrarily shaped subdiffraction features with a propagation distance of 250 Rayleigh lengths have been demonstrated by the superposition of optical Bessel beams of different orders but the same transverse wavenumber^131^. An ultralong subdiffraction diffractionless beam was generated by an SLM with 256 phase levels in the range of 0‒2π. The beam achieved a propagation distance of approximately 43.3 mm with a maximum transverse size of less than 63.28 μm (0.5λ/NA) for a working distance of 1000 mm^132^. Generating subdiffraction and subwavelength diffractionless beams using an SLM and a high-resolution imaging system is also possible. Another promising method is to use phase-modulation birefringent metasurfaces^105–107^ for the direct generation of diffractionless beams with complex polarization in broadband wavelength ranges.

### Generation of subdiffraction three-dimensional (3D) hollow spots

Unlike 2D hollow spots, 3D hollow spots provide complete confinement in 3D space, which can be used to increase the axial resolution in STED microscopy^92^ and superresolution lithography^93^ and improve the trapping stability of optical tweezers. Different approaches have been proposed for the generation of subdiffraction or subwavelength 3D hollow spots, including focusing of radially polarized first-order Laguerre-Gaussian waves with a 4π system^133^, focusing of circularly polarized waves with a circular π phase plate (πPP)^134^, destructive interference of double-ring-shaped radially polarized R-TEM11*-mode waves^135^, incoherent superposition of two radially polarized waves that are modulated by a circular πPP and a quadrant 0/π phase plate^136^ and beam shaping with an SLM^137^. A 3D hollow spot was also demonstrated in visible-light via antiresolution^138^. However, the 3D hollow spots that are generated by these methods are either diffraction limited^134–138^ or difficult to realize^133^.

**Fig. 8:**
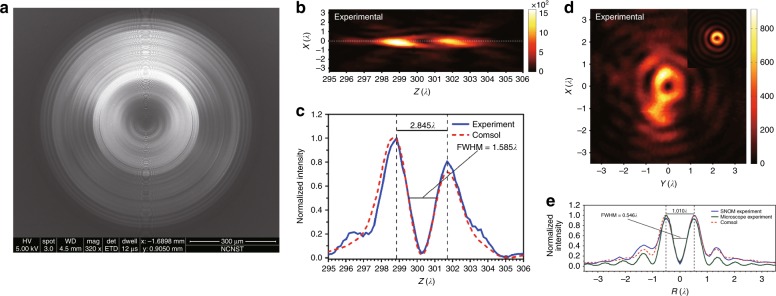
Generation of a subdiffraction 3D hollow spot. **a** An SEM image of a lens for the generation of a 3D hollow spot; **b** the 2D optical intensity distributions on the propagation plane; **c** a comparison of the axial intensity distribution curves between the theoretical and experimental results; **d** the 2D optical intensity distributions on the focal plane; and **e** a comparison of transverse intensity distribution curves between the theoretical and experimental results on the focal plane. Reproduced with permission from ref. ^139^ Copyright 2018, The Optical Society of America

A SOL based on a BP concentric ring array was designed by carefully optimizing the interference patterns of the azimuthal, radial and longitudinal polarizations in cylindrically polarized vector waves. Since the azimuthal and radial components share the same transmission function, a tradeoff must be made between the transverse and axial sizes. As shown in Fig. 8, a 3D hollow spot was experimentally created with a transverse inner FWHM of 0.546λ (0.496λ/NA) and an axial inner FWHM of 1.585λ at a wavelength of 632.8 nm^139^. Further investigation showed that both the transverse and axial sizes can be significantly reduced by independently modulating the radial and azimuthal components of the incident waves using birefringent metasurfaces. Numerical simulations have demonstrated the generation of a 3D hollow spot with inner FWHMs of 0.33λ and 1.32λ in the transverse and axial directions, respectively, in air.

### Broadband and achromatic focusing

Thus far, most reported SOLs have been for monochromatic operation. Broadband and achromatic performances are highly desirable in practical applications. Utilizing the broadband phase-tuning property of plasmonic dipole antennas, a metallic nanoaperture array was employed to construct a broadband focusing lens based on BP (0, π) modulation by rotating the nanoaperture by angles of 0 and π/2. The plasmonic metasurface lens demonstrated ultrabroadband focusing with a spot size of 0.662–0.696 times the diffraction limit, which is smaller than the superoscillation criterion, in the wavelength range of 405–785 nm. However, the sidebands were 5 times stronger than the central peak^140^. Broadband subdiffraction focusing can also be realized with BP lenses consisting of dielectric concentric rings. Our numerical simulations have shown subdiffraction focusing of radially polarized waves in a wavelength range of 632.8–680 nm, and the focal spot has an FWHM of ~0.399λ–0.452λ with a small sidelobe intensity of less than 16.2% of the intensity of the central spot. Recently, a dielectric metalens was proposed that is based on a group of 30 dielectric half-wave metasurfaces with equally spaced phase values in the 2π range. The metalens integrates the functions of polarization conversion (LP to RP) and phase modulation. According to a numerical simulation, with a hyperbolic phase profile and extra π phase modulation, the proposed lens can generate subdiffraction focal spots over a wide wavelength range of 875–1025 nm^107^. Although achromatic operation was demonstrated with an SOL at three wavelengths, i.e., 405, 532 and 63, broadband achromatic subdiffraction focusing remains challenging^141^. The possibility of broadband achromatic subdiffraction focusing was recently considered for two small metamirrors with a hyperbolic phase profile, which were made of 32 metallic cross-bar quarter-wave structures for independent manipulation of the polarization and the phase. Achromatic subdiffraction and superoscillation focusing of radially and azimuthally polarized waves was verified by numerical simulation with broadband wavelength ranges of 100 and 210 nm, respectively^106^. However, broadband achromatic superoscillation focusing by a large-aperture and high-NA lens has not yet been demonstrated. Although achromatic planar metalenses have been reported using dispersion compensation^142–144^, the corresponding design method cannot be simply applied to superoscillation focusing devices because superoscillation relies on delicate interference of the propagation waves.

### Quantum optical superoscillation

Previous demonstrations of optical superoscillation have been reported in the domain of classical optics, in which the optical superoscillation was associated with the interference of classical propagating waves. Owing to the wave-particle duality, superoscillation is expected at the level of single photons, which are described by the quantum wavefunction. Experimental observation of single-photon superoscillation with a conventional 1D SOL has recently been reported^145^. Similar to multiple-slit interference, a 1D SOL that is based on a binary mask was designed with superoscillation focusing performance for classical optical interference. In the experiment, a single-photon source was used to study the superoscillation behaviour of a single photon passing through a grating-like binary mask. The measured single-photon wavefunction was confined to an area with FWHMs of (0.49 ± 0.02) λ and (0.48 ± 0.03) λ for two orthogonal polarizations, which are larger than both the theoretical and experimental values of 0.4λ and 0.44λ in the classical regime. The superoscillatory behaviour of a single photon indicates that optical superoscillation is not a group behaviour but rather a natural result of quantum behaviour^145^. Table 1 summarizes reported results for superoscillatory lenses.

## Design methods

### Optimization design methods

**Fig. 9:**
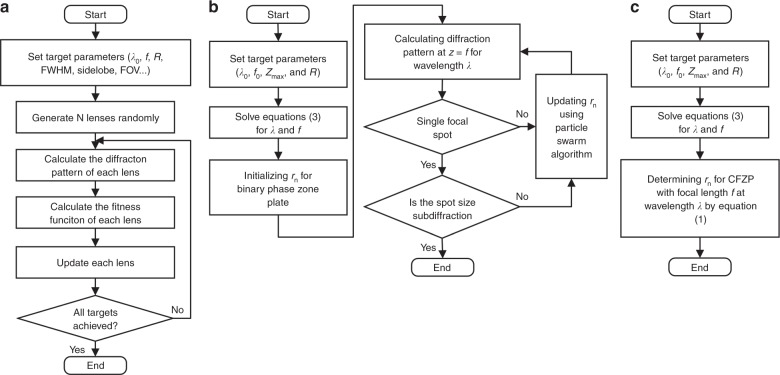
Design methods of superoscillatory lenses. **a** A particle swarm algorithm for SOL optimization; **b** the subdiffraction diffractionless beam optimization procedure; **c** the optimization-free design of a subdiffraction diffractionless beam. Equations (3) is given by *f* = *z*(0) and *Z*_max_ = *z*(*R*)−*z*(0), where *z*(*r*) = {1−[λ_0_/d(*r*)]^2^}^1/2^, d(*r*) = λ/sin(θ(*r*)), θ(*r*) = atan(*r*/*f*), *R* is the radius of the lens, *Z*_max_ is the beam propagation distance, λ_0_ and *f*_0_ are the working wavelength and focal length, and λ and *f* are the designing wavelength and focal length. Equation (1) is given by *r*_n_ = [*n*λ*f* + (*n*λ)^2^/4]^1/2^, where n is an integer. Reproduced with permission from ref. ^130^ Copyright 2018, The Optical Society of America

The design of an SOL mainly relies on optimization algorithms, among which particle swarm algorithms^146^ are the most commonly used. The design procedure is described in the flow chart in Fig. 9a. First, for specified parameters of the target field, such as the FWHM, sidelobe, FOV and DOF, a group of lenses with different genes representing the lens transmission function are randomly generated. Then, the diffraction pattern of each lens is calculated on the target focal plane for specified incident waves. By comparing the diffraction and target fields, a fitness function is calculated for each lens. Finally, the gene of each lens is updated according to the fitness function. This procedure is repeated until the best fitness function attains a predefined value. With an optimization approach, SOLs can be designed without fully understanding the physics behind superoscillations; however, the particle swarm algorithm might fall into a locally optimal solution. This problem can be partially alleviated by randomly regenerating the genes of some of the particles after certain iterations. The solution can also be improved by combining the particle swarm algorithm with genetic algorithms^147^. Based on genetic algorithms, an unconstrained multi-objective optimization method^148^ was proposed to realize flexible focusing patterns. The methods for diffraction pattern calculation include the Rayleigh-Sommerfeld approach^149^, the angular spectrum method^150^ and the Debye-Wolf method^151^. Due to the computational complexity of the 2D integration involved in the calculation, the size of the lens under design is restricted to several hundreds of working wavelengths. However, in the case of a circular symmetry configuration^65,86,88,95^, the calculation can be simplified to 1D integration, which can be further accelerated by utilizing the fast Hankel transformation^152^. Nevertheless, the design of larger aperture lenses for subwavelength and superoscillation focusing remains a substantial challenge.

### Optimization-free design approaches

**Fig. 10:**
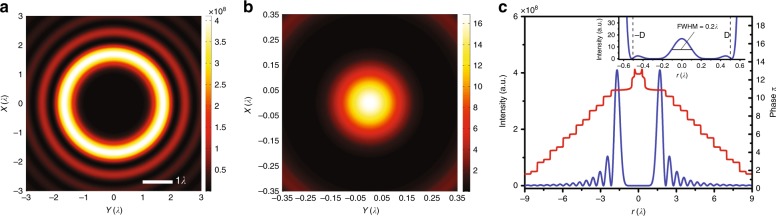
Example of superoscillatory spot constructed from band-limited CPSWFs φ_n_(*c*,*r*), where *c* = 2π*D*/λ, the cut-off frequency is 2π/λ and the FOV is *D* = λ/2. **a** The 2D intensity distribution of the constructed superoscillatory spot that is surrounded by very large sidebands; **b** A magnified image of the intensity-constructed superoscillatory spot; **c** The intensity distribution curve (blue) and phase distribution curve (red) in the radial direction: the inset presents the intensity distribution curve within the FOV [−*D*, *D*], which shows an FWHM of 0.2λ

Although convenient, optimization methods provide a very coarse physical picture of optical superoscillation and do not enable control of the detailed profile of the superoscillatory field. Moreover, for limited target parameters, the solution obtained via an optimization method is not unique, and tradeoffs must be made among the target parameters for multiparameter optimization. Since optical superoscillations are induced by the interference of coherent propagation waves or the superposition of plane waves with spatial frequencies that are less than 1/λ, the superoscillation optical field can be described with bandwidth-limited functions. PSWFs constitute a complete set of 1D bandwidth-limited functions and their properties have been thoroughly studied in a series of papers^49,153–156^ by D. Slepain, H.J. Landau and H. O. Pollak. The PSWFs with a bandwidth of *k*_*0*_ are orthogonal in both the entire spatial domain and the limited region of [−*D*/2, *D*/2]. This property allows one to synthesize arbitrarily narrow structures in 1D space. Based on PSWFs, an optimization-free approach was proposed to construct a superoscillation focal spot for a given optical field profile and FOV [−*D*/2, *D*/2], and the corresponding superoscillatory mask transmission function could be obtained by reverse propagation using the scalar angular spectrum method^50^. This approach can also be extended to 2D cases for optimization of the superoscillatory point spread function (PSF) for far-field superresolution imaging^157^ using circular prolate spheroidal wavefunctions (CPSWFs)^158^. Figure 10 presents an example of a superoscillatory function that was constructed from band-limited CPSWFs *φ*_n_(*c*, *r*), where *c* = 2π*D*/λ, the cut-off frequency is 2π/λ and FOV is *D* = λ/2. The constructed superoscillatory function has an FWHM of 0.2λ. Theoretically, this method is reported to improve the efficiency by three orders of magnitude for the same resolution or increase the resolution by 26% for the same efficiency^157^. The superoscillatory masks that are designed with band-limited functions typically have continuous amplitude and phase distributions with phase shifts at points of zero amplitude.

An optimization-free mathematical method for designing a superoscillatory mask by solving a nonlinear matrix equation was proposed^58^. For target intensity values [*f*_1_, *f*_2_,…, *f*_M_] at radii [*r*_1_, *r*_2_, …, *r*_M_] on the focal plane and a fixed ring belt width Δ*r* on the mask, the radius of the *n*th belt was obtained by solving the inverse problem with trust-region dogleg Newton theory^58^.

### Design of optical needles and diffractionless beams

Various methods have been employed to design SOLs of long DOF, including optical needles and diffractionless beams. A prescribed super-Gaussian function can be used to describe the extended longitudinal profile of an optical needle. Using optimization algorithms, the transmission function of a BA mask can be obtained by minimizing the difference between the actual field distribution and the merit function^114–117,124,159^. However, when applying these commonly used methods, one must calculate the diffraction patterns on the planes at different positions within the target optical needle at intervals that are smaller than one wavelength and then compare the calculated patterns with their merit profiles. Therefore, the required computational consumption increases linearly with the length of the optical needle, which renders designing subdiffraction optical needles with propagation distances that exceed several tens of wavelengths impractical. An optimization-free approach was proposed for the generation of a longitudinally polarized optical needle^160^, which can be treated as a constant electric current within its extent. Reverse propagation was carried out to obtain the profile of a radially polarized incident beam at the pupil plane of the two high-NA objective lenses in a 4Pi system. However, this approach cannot be extended to cases other than longitudinal polarizations.

According to angular spectrum theory, the profile of a propagating optical field is determined by its angular spectrum. Due to a property of propagating waves in a uniform lossless medium, the amplitude of the angular spectrum remains unchanged during wave propagation. The variation in the transverse field distribution results solely from the accumulated phase difference for spatial frequency components. Therefore, the key to generating a subdiffraction diffractionless beam is to synthesize the optical field of the subdiffraction spot on a given plane while minimizing the accumulation of the phase difference over a desired propagation distance. This can be done by reducing the propagation angle with respect to the optical axis for each spatial frequency component or by compressing the angular spectrum with respect to the effective cutoff spatial frequency of the propagating wave. As shown in Fig. 9b, utilizing the concept of angular spectrum compression^129^, an SOL was designed with a single subdiffraction focal spot at wavelength λ. Then, under illumination at a shorter wavelength λ_0_ (<λ), a subdiffraction diffractionless beam was generated with a length of approximately 100λ_0_ in air and 200λ_0_ in water. The value of the wavelength λ is determined by several parameters, including the lens radius, the working wavelength λ_0_, the focal length and the optical needle propagation distance. The extension of an optical needle in water^124,129^ can be explained similarly. Interestingly, utilizing the same strategy, subdiffraction diffractionless beams can be created with the same transverse size and propagation distance but lower intensity fluctuations along the optical axis using a classical Fresnel zone plate, which is entirely free from optimization and allows one to design subdiffraction diffractionless beams for multiple polarizations using very simple algebra^130^, as shown in Fig. 9c. The main shortcoming of angular spectrum compression is that it enables little control over the detailed field profile within the propagation distance. Further investigation is necessary for generating uniform diffractionless beams with superoscillatory transverse size.

## Characterization of superoscillatory optical fields

### Transverse fields

**Fig. 11:**
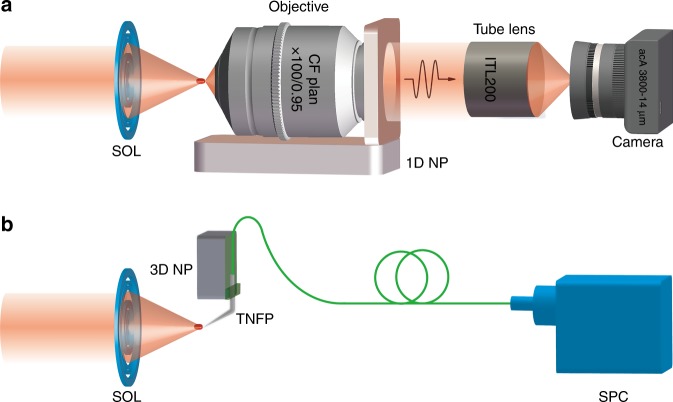
Superoscillation optical field characterization systems. **a** The superoscillatory fields are obtained by a high-NA microscope with an objective lens mounted on a 1D nanopositioner (1D NP), where the camera is used to obtain the 2D intensity distribution of the diffraction pattern. **b** A testing system based on a tapered nanofibre probe (TNFP) mounted on a 3D nanopositioner (3D NP), where a single photon counter (SPC) is used to detect the weak optical signal from the nanofibre probe

Optical superoscillatory features result from the interference of propagation waves, whose angular spectrum is restricted within the cutoff spatial frequency of 1/λ. Therefore, ideally, superoscillatory features can be retrieved by an optical system with an NA of one and an infinite aperture. As shown in Fig. 11a, high-NA microscopes have been widely used to characterize superoscillatory optical fields^61,85,96,114,116,124,129,130^ and the 2D intensity distribution of a superoscillatory optical field can be directly obtained in a single shot by a conventional optical microscope equipped with a high-resolution digital camera. In addition, 3D mapping of superoscillatory optical fields can be implemented by scanning an objective lens mounted on a *z*-axis piezo-driven nanopositioner. The major advantage of using microscopy is its fast measurement. However, the pixel size is quite large compared to the wavelength in the visible and near-infrared spectra. To resolve the images of superoscillatory fields, microscopes with large magnification must be employed. Both theoretical and experimental results have indicated that this optical lever results in significant attenuation of the longitudinal component in the image field^64^. The absence of longitudinal polarization was verified in later experimental investigations of focused superoscillation optical fields^139,161^, which showed clear deviations of image fields from the theoretically predicted electric fields and satisfactory agreement was observed between the image fields and the transverse components that were obtained via numerical simulation.

### Longitudinal fields

In addition to conventional microscopes, a scanning aperture optical microscope operated in transmission mode was applied to map focused superoscillation spots^60^. The light can be collected through a metal-coated tapered fibre tip with an aperture of as small as 30 nm. The polarization filtering behaviour of such tips has been theoretically studied using an electric dipole scattering model^64^ and high polarization selectivity with a substantial reduction in the polarization parallel to the tip axis is observed. As shown in Fig. 11b, to measure the longitudinal electric components, the fibre tip axis must be set perpendicular to the polarization direction or at an angle at which the tip can respond to the longitudinal polarization to a certain extent^88,130^. A titled tip can be used to map the entire electric field within the plane of a tilted nanofibre with satisfactory similarity to the theoretical result of the entire field^130,139^, and the distortion that is caused by the polarization selectivity can be minimized with a tilt angle of 45°.

### Vector fields

In addition to the above two direct approaches for characterizing superoscillatory fields, another way to obtain the intensity distribution of the optical field is to use the knife-edge method^162^. The advantage of using the knife-edge method is its insensitivity to polarization, which makes it suitable for characterizing the entire optical field components. By projecting in different directions, the data acquired with a single knife edge can be used to reconstruct the 2D intensity distribution via Radon back-transformation^163^. Instead of traditionally used razor blades, the knife edge can be formed by a sharp-edged opaque pad that is deposited on the top surface of a photodiode active area with minimized edge diffraction effect. The experimental results demonstrated that the reconstructed subdiffraction field profiles had excellent agreement with the theoretical results of total fields, indicating that the method has satisfactory polarization insensitivity^72^. A similar approach was utilized to characterize a subdiffraction focal spot with a size of 0.4λ that was generated by focusing a radially polarized wave at a wavelength of λ = 980. In the experiment, a specially designed detector was used. A 200-nm-thick Ti/Au knife edge with a roughness of less than 30 nm was formed on the top surface of a 200-nm-thick and 50 μm × 25 μm active area, which was fabricated on the top surface of a depletion layer. The experimental results showed good agreement with the theoretical results^119^. In the above cases, only one knife edge was involved in the scanning, and the 2D intensity distribution was recovered with Radon back-transformation. This postprocessing step can be avoided by using a double knife edge with the edges oriented in the *x*- and *y*-directions^164^, which allows the calculation of the intensity within a smaller area that is determined by the scanning interval in the x- and y-directions via simple subtraction operations. A double knife edge was fabricated from a right-angled silicon fragment with a thickness of 110 μm and a roughness of 10 nm and it was directly mounted on a conventional photodetector. Due to the high reflectivity of silicon, the measurement could be conducted in both the reflection and transmission modes^165^. Due to the diffraction effect that is caused by the 110-μm-thick edge, the measured subdiffraction spots showed clear distortion compared to their theoretical predictions^166^. This discrepancy might be minimized by a double knife edge with nanometre-scale thickness that is deposited directly on the top surface of the photon detector, as reported in the literature^72,119^.

### Phase retrieval

Phase retrieval is important for understanding the generation mechanism of optical superoscillations, as gigantic local wavevectors are known to be closely associated with the formation of superoscillations^57^. To experimentally obtain the phase distribution of superoscillation optical fields, a monolithic metamaterial interferometer with a superoscillatory Pancharatnam-Berry phase metasurface was proposed. The interferometer consists of rows of subwavelength metallic slits oriented in either the +45° or −45° direction, which results in a 0 or π phase shift for cross-polarized transmission waves. Such a metasurface has a negligible effect on the phase distribution of the transmitted waves that have the same polarization as the linearly polarized incident waves; therefore, this copolarized transmission wave can be used as a reference for interference with the phase-modulated cross-polarized wave. A one-dimensional superoscillation focusing lens was designed by utilizing the BP, i.e., 0 and π, modulation mechanism of the metasurface; therefore, the reference and superoscillation waves were simultaneously created through such a metasurface interferometer without any moving parts. Phase reconstruction was performed with polarization-dependent intensity measurements for incident waves with left- and right-handed CPs and LPs oriented at angles of 45° and −45°. The interference patterns were collected by a conventional microscope with a high NA and a large magnification. Four characteristic features of the superoscillatory field, namely, a high localized electric field, phase singularities, gigantic local wavevectors and energy backflow, were extracted via this technique, with a resolution of λ/100. However, this phase mapping approach is difficult to extend to more complicated cases. Moreover, the full retrieval of the phase and amplitude for individual polarization components remains challenging.

## Applications

### Superoscillation imaging

**Fig. 12:**
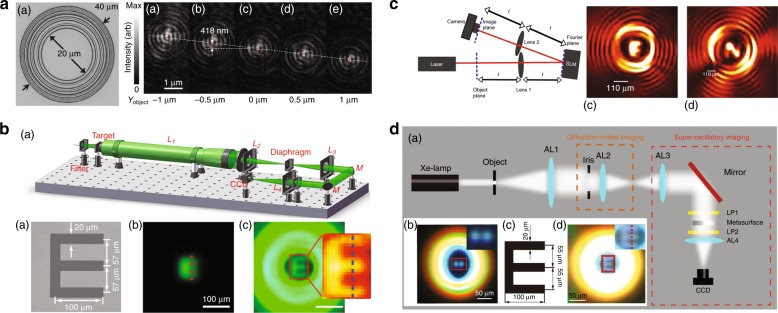
**Superresolution imaging via superosicllation. a** An SEM image of an optical needle super-oscillatory lens (ONSOL) for sub-diffraction imaging and the experimental results of point-source imaging. In the imaging experiments, the point image shifts when the ONSOL moves by 1μm on either side of central position. In (a)–(e), the hotspots measure 0.48λ, which is smaller than the diffraction limit. Reproduced with permission from ref. ^168^ Copyright 2014, AIP Publishing. **b** A schematic diagram of a super-resolution telescope, including a halogenated lamp, a filter, an optical collimator (L1), an entrance pupil, an objective lens (L2) and a 4ƒ system consisting of a field diaphragm, two mirrors (M), two focusing lenses (L3 and L4), a designed phase plate and a CCD camera, and its imaging experimental results: (a)-(**c**) A microscope image of an “E” target, an experimental diffraction-limited imaging pattern and a super-resolution imaging pattern. Reproduced with permission from ref. ^169^ Copyright 2016, NPG, under a Creative Commons license (https://creativecommons.org/licenses/by/4.0/). **c** A schematic diagram of the experimental setup for superresolution far-field imaging of complex objects using reduced superoscillating ripples and the superresolution imaging of letters “F” and “N”. Reproduced with permission from ref. ^171^ Copyright 2017, The Optical Society of America. **d** Broadband achromatic superoscillation imaging using a superoscillatory pupil filter: **a** the setup of a 4f system with conventional achromatic lenses AL, AL2, AL3 and Al4 and a superoscillatory pupil filter metasurface; **b** subdiffraction imaging of two holes; **c** a complex object “E” and **d** its superresolution image. Reproduced with permission from ref. ^172^ Copyright 2010, John Wiley and Sons

The imaging properties of an SOL that is based on a Penrose nanohole array have been examined with a single point source and multiple point sources both theoretically and experimentally for coherent and incoherent illumination^167^. Incoherent illumination resulted in a higher resolution of 450 nm at a wavelength of 660 nm. The study also suggested that the 200 × 200 μm^2^ nanohole array can achieve an imaging resolution that is comparable to that of a conventional lens with a high NA, while the imaging resolution remains larger than the diffraction limit. Theoretical research was also carried out to investigate the imaging performance of a 1D SOL designed with band-limited functions^150^. Numerical simulations showed that two 0.04λ-wide slits that were separated by 0.24λ were imaged on the plane 20λ from the lens. Even in the presence of the very large sideband in the lens PSF, according to the Rayleigh criterion^163^, the two slits can be well resolved within the lens FOV under incoherent illumination. The imaging capabilities of an optical needle SOL based on binary concentric rings were experimentally studied^168^. In the experiment, the object and image distances were 39λ and 14λ, respectively. The results showed a PSF with an FWHM of 0.38λ for on-axis imaging, which is 24% smaller than the diffraction limit, and an effective NA of 1.31 in air at a wavelength of 640 nm. For off-axis imaging with an object displacement of 1.56λ, the measured image spot size was 0.48λ, which is smaller than the diffraction limit. Superoscillation imaging was also used to improve the resolution of an optical telescope system through a carefully designed pupil filter, which allows superresolution imaging within a small local FOV that is restricted by the large neighbouring sideband^169^. Generally, direct superoscillation imaging is limited by the very large sideband that surrounds the superoscillatory region in the PSF. To suppress the sideband and increase the sensitivity of the superresolution imaging system, for the 1D case, the concept of selective superoscillation was suggested for producing a fast oscillation region of a superoscillation waveform while avoiding high-energy content^170^. In the case of 2D imaging, a new class of superoscillation functions was proposed for designing a superresolution PSF with a subdiffraction spot surrounded by superoscillatory ripples of low intensity. In this way, the sideband energy is significantly suppressed, which allows one to expand the image area and therefore improve the imaging sensitivity. The corresponding experimental demonstration was conducted with a 4F imaging system^171^. Recently, broadband superresolution imaging was achieved with an improved resolution of 0.64 times the Rayleigh criterion by using a 4F system that was composed of four conventional achromatic lenses and a broadband superoscillatory pupil filter, which consists of grating-shape metallic metasurfaces for BP modulation^172^. Recent superresolution imaging results that were obtained by utilizing superoscillatory PSFs are summarized in Fig. 12.

### Superresolution microscopy

#### Superresolution microscopy based on an SOL

**Fig. 13:**
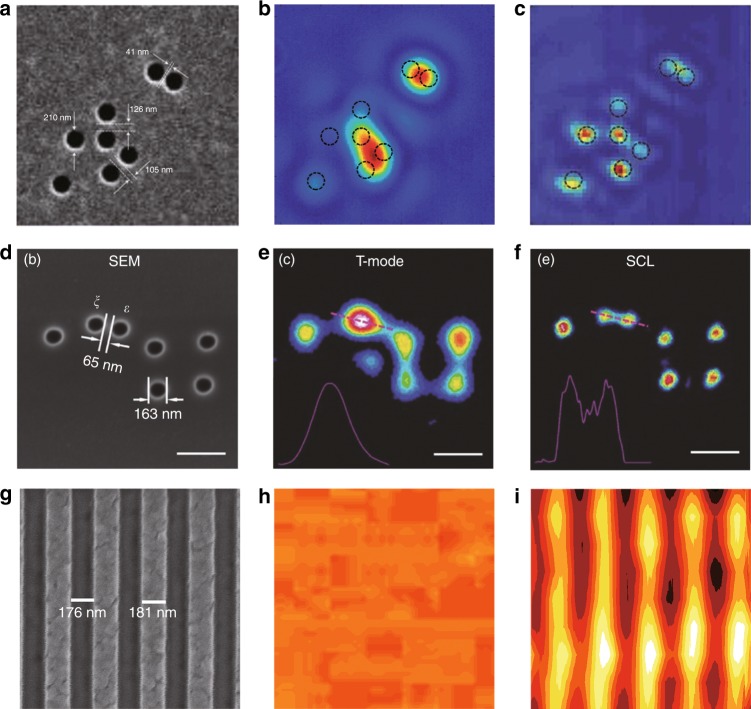
Superresolution microscopy based on superoscillatory lenses. **a** An SEM image of a cluster of 210 nm nanoholes in a metal film. **b** An image of the cluster obtained with a conventional microscope with NA = 1.4. **c** An SOL image of the nanohole cluster (dashed circles map the positions of the holes). Reproduced with permission from ref. ^62^ Copyright 2012, Springer Nature. **d** An SEM image of a cluster of 163 nm nanoholes in a metal film. **e** An image of the cluster obtained with a laser scanning confocal microscope. **f** An SCL image of the nanohole cluster. Reproduced with permission from ref. ^121^ Copyright 2017, John Wiley and Sons. **g** An SEM image of a subdiffraction grating with a linewidth of approximately 180 nm. **h** An image of the grating obtained with a conventional microscope with NA = 0.9. **i** A diffractionless SOL image of the grating obtained in the confocal setup with a conventional microscope with NA = 0.9

One of the most promising applications of superoscillation is label-free far-field superresolution microscopy. Direct imaging with SOLs is restricted by the limited FOV. This problem can be overcome by the confocal configuration in which a superoscillation hot spot is used as a point illumination source. The sideband effect can be significantly suppressed because the system PSF is the product of the PSFs of the SOL and objective lens. Figure 13 shows cases of superresolution microscopes that are based on SOLs.

**Table 1:** Reported results for superoscillatory lenses

Type	Refs.	FWHM (λ)	Ambient medium	DOF (λ)	Focal length (λ)	NA	Wavelength (λ)	Polarization	DL^a^ (λ)	SOC^b^ (λ)
Focusing linearly and circularly polarized waves	Wang et al.^61^	0.6	Air	4.51	5.26	0.79	532 nm	–	0.63	0.48
	Rogers et al.^62^	0.289	Oil	–	16.1	1.35	640 nm	–	0.37	0.28
	Li et al.^63^	0.271^c^	Oil	–	1.88^c^	1.507	532 nm	LP	0.33	0.25
	Chen et al.^65^	0.454	Air	–	399.5	0.78	632.8 nm	CP	0.64	0.487
	Liu et al.^66^	0.46 (x-axis) 0.45 (y-axis)	Air	1.1	60.74	0.95	532.4 nm	CP	0.53	0.40
	Wan et al.^67^	0.49^c^	Air	–	100^c^	0.82	400 nm	CP	0.61	0.46
	Chen et al.^68^	0.379	Air	–	41.9	0.976	632.8 nm	LP	0.513	0.39
	Chen et al.^69^	0.406	Air	–	148	0.936	632.8 nm	LP	0.534	0.406
	He et al.^70^	0.319^c^	Air	–	20^c^	0.996	365 nm	LP	0.50	0.38
	Huang et al.^71^	0.316	Air	–	21	0.83	632.8 nm	LP	0.60	0.458
Focusing cylinderically polarized vector waves	Venugopalan et al.^83^	0.41	Air	–	0.948 1.6	0.71	632.8 nm 750 nm	RP	0.704	0.535
	Liu et al.^85^	0.42 (x-axis) 0.52 (y-axis)	Water	0.92	1.49	0.91	808 nm	RP	0.55	0.418
	Liu et al.^86^	0.25^c^	Oil	–	347.5^c^	1.42	532 nm	RP	0.35	0.27
	Ye et al.^87^	0.39^c^	Air	–	16.3^c^	0.94	632.8 nm	RP	0.53	0.40
	Yu et al.^88^	0.456	Air	5	200	0.93	632.8 nm	RP	0.537	0.409
	Chen et al.^95^	0.61	Air	–	599.8	0.64	632.8 nm	AP	0.781	0.594
	Wu et al.^96^	0.368	Air	2	200.89	0.955	632.8 nm	AP	0.523	0.398
	Luo et al.^104^	0.47^c^	Air	–	5.8^c^	0.86	857 nm	RP	0.58	0.44
		0.43^c^	Air	–	5.8^c^	0.86	857 nm	AP	0.58	0.44
	Li et al.^106^	0.41^c^	Air	–	2^c^	0.96	1550 nm	RP	0.52	0.395
		0.34^c^	Air	–	2^c^	0.96	1550 nm	AP	0.52	0.395
	Zuo et al.^107^	0.419^c^	Air	5.6^c^	6.56^c^	0.956	915 nm	RP	0.52	0.397
Sub-diffraction optical needles and diffractionless beams	Rogers et al.^113^	0.42	Air	11	9.2	0.96	640 nm	LP	0.521	0.396
	Yuan et al.^114^	0.45	Air	15	24.7	0.89	405 nm	CP	0.529	0.425
	Liu et al.^115^	0.67^c^ (x-axis) 0.31^c^ (y-axis)	Water	12.4^c^	75^c^	1.25	193 nm	LP	0.40	0.31
	Qin et al.^116^	0.42–0.49	Air	12	240	~0.95	633 nm	AP	~0.52	~0.40
	Ruan et al.^117^	1.212	Air	19.7	420	~0.36	118.8μm	LP	~1.41	~1.07
	Wang et al.^118^	0.43^c^	Air	4^c^	–	0.95	–	RP	0.526	0.40
	Kitamura et al.^119^	0.4^c^	Air	4^c^	–	0.9	980 nm	RP	0.556	0.422
	Peng et al.^120^	0.44^c^	Air	2.65^c^	2.12^c^	0.81	632.8	RP	0.62	0.47
	Qin et al.^121^	0.407	Air	12	135	0.98	405 nm	CP	0.51	0.39
	Chen et al.^124^	0.34–0.52	Air	10	300	0.91	632.8 nm	AP	0.55	0.418
	Zhang et al.^129^	0.34–0.53	Air	94	~230	~0.93	632.8 nm	AP	0.538	0.408
	Wu et al.^130^	0.40–0.54	Air	90	~230	~0.93	632.8 nm	CP	0.538	0.408
		0.43–0.54	Air	73	~230	~0.93	632.8 nm	RP	0.538	0.408
		0.34–0.41	Air	80	~230	~0.93	632.8 nm	AP	0.538	0.408
	Wu et al.^132^	94–98	Air	68,426	1585000	0.005	632.8 nm	LP	100	76
Generation of sub-diffraction 3D hollow spots	Wu et al.^139^	0.546 (Lateral) 1.585 (Axial)	Air	–	300	0.91	632.8 nm	RP&AP	0.55	0.42
Broadband and achromatic	Tang et al.^140^	2.17 1.65 1.39 1.18	Air	–	229.6 133.5 94.8 61.1	0.16 0.21 0.24 0.30	405 nm 532 nm 632.8 nm 785 nm	CP	3.14 2.42 2.06 1.68	2.39 1.84 1.57 1.27
	Yuan et al.^141^	0.457 0.445 0.54	Air	–	24.69 18.80 15.80	0.89	405 nm 532 nm 633 nm	LP	0.56	0.43
Optical quantum superoscillation	Yuan et al.^145^	0.49	Air	–	12.3	0.95	810 nm	CP	0.53	0.40

*LP* linear polarization, *CP* circular polarization, *RP* radial polarization, *AP* azimuthal polarization

^a^Diffraction limit, 0.5λ/NA

^b^Superoscillation criteria, 0.38λ/NA

^c^Theoretical results, experimental othervise

The first demonstration^62^ of this technique was performed with a conventional liquid immersion microscope with an NA of 1.4. At a wavelength of 640 nm, the SOL generated a hot spot with an FWHM of 185 nm at a distance of 10.3 μm. The neighbouring sidelobe had the same intensity as the hot spot and the separation between them was approximately 200 nm. By scanning the sample using a 2D nanotranslation stage, a superresolution image of nanoholes fabricated on a 100-nm-thick titanium film was obtained. Two 210-nm-diameter nanoholes spaced 41 nm apart were nearly resolved, as shown in Fig. 13c.

To further increase the working distance and reduce the sidelobe intensity, a critical superresolution lens was developed^121^ with a focal length of 135λ, a transverse size of 0.407λ and a DOF of 12λ at a wavelength of 405 nm. Similarly, two nanoholes with diameters of 163 nm and a spacing of 65 nm could be well resolved using the confocal configuration with a conventional microscope with an NA of 0.7, as shown in Fig. 13f. The advantage of using a long-DOF SOL is that images of structures of different heights can be simultaneously acquired in a single 2D scan. Although the DOF is longer compared to the previous work, it is still too short to penetrate samples in most practical applications.

Self-reconstructing beams, such as Bessel beams, are promising candidates for deep penetration microscopy. Theoretical and experimental studies have been carried out to study the microscopy technique with Bessel beams, which show unexpected robustness against deflection at object surfaces. This approach not only reduces scattering artefacts but also increases the image quality. Moreover, it allows penetration deep into dense media^173^. Recently, based on a diffractionless SOL^129,130^, a superresolution image of a subwavelength metallic grating with a linewidth of 0.28λ was obtained with a visibility that exceeded 20% at a working wavelength of 632.8 nm, as shown in Fig. 13i. Unlike previously reported cases, the diffractionless superoscillation beam can penetrate a 175-μm glass plate and realize superoscillation illumination on the metallic grating that was fabricated on the top surface of the glass plate. Because of the excellent penetration and superoscillatory transverse size, this method is suitable for practical use. More importantly, it might enable label-free 3D superresolution imaging of samples.

#### Superresolution microscopy based on an SLM

An SOL with a fixed amplitude or phase mask can only be applied to a normally incident wave. Without wide-angle superoscillation focusing performance, superresolution microscopes based on SOLs must operate in raster scan mode and their imaging speed is restricted by the scanning speed of the piezo translation stages, with which achieving real-time imaging is difficult. SLMs provide a flexible way to design a reconfigurable superoscillatory focus with real-time speed. In a 4F imaging system with an NA of 0.00864, a reflective SLM is located on the Fourier plane of the 4F system, where the SLM acts as a spatial filter to form a superoscillatory PSF for a working wavelength of 632.8 nm. Although the superoscillatory spot is surrounded by large sidelobes, superresolution imaging can be realized with a resolution of 72% of the diffraction limit (36.7 μm) for objects located within the FOV of 150 μm^174^. Recently, real-time subwavelength superresolution based on two SLMs was reported by Rogers et al. of University of Southampton. The system was a modification of a standard confocal microscope in which two SLMs were applied to shape the laser beam as it entered the microscope. In addition, polarization measurements were taken to form a polarization contrast superresolution image, which revealed new levels of information in biological samples. In addition to label-free microscopy, superoscillation focal spots were utilized to enhance the spatial resolution of confocal laser scanning microscopy^175^.

#### Other applications

To achieve high-density data storage, a superoscillation focusing device with a long DOF was suggested and experimentally explored as an alternative technique, which can focus light into sub-50 nm spots with a DOF of 5λ in magnetic recording material at a wavelength of 473 nm^176^. In addition to applications in the spatial domain, the concept of superoscillation can also be applied to the time domain for optical pulse shaping to break the temporal resolution limit. Temporal features with a duration of 87 ± 5 femtoseconds, which is three times shorter than that of a transform-limited Gaussian pulse, have been experimentally demonstrated with a visibility of 30%^177^. To achieve arbitrarily short features, generic methods have been proposed and demonstrated for synthesizing femtosecond pulses based on Gaussian, Airy and Hermite-Gauss functions^178^. Such superoscillatory pulses might be promising for ultrafast temporal measurements. The concept of superoscillation can also be extended to its complementary counterpart, suboscillation, in which a local arbitrarily low frequency can be realized with a lower-bound-limited function, which can be used for optical super defocusing^179^.

## Conclusions

In summary, recent developments in the area of optical superoscillations have shown great potential for superresolution optical focusing and imaging. Optical devices to realize subdiffraction and subwavelength focusing of optical waves with different polarizations have been demonstrated. Vector optical fields with special diffraction patterns, including subdiffraction diffractionless beams of different polarizations and 3D hollow spots, have been experimentally demonstrated. The applications of superoscillation in telescopes have shown improved resolution beyond the traditional resolution limit. Microscopes based on superoscillatory devices, including point superoscillation focusing lenses, long-DOF supercritical lenses and superoscillation diffractionless lenses, have been achieved with advantages of noncontact, far-field and label-free operation. In addition, superoscillation focusing has been applied to improve the resolution of superresolution microscopy based on fluorescent labels. Ultrahigh-density optical data storage was also demonstrated with superoscillatory optical needles. Applying metasurfaces to superoscillatory optical devices enables flexible control of the phase and the polarization to achieve complex superoscillatory optical fields for specific applications. Many challenging issues remain, the most important of which is the efficiency: If the spot size is far below the superoscillation criterion value, there is an exponential decrease in the efficiency. In all reported cases, the focused waves within the FOV only constitute a few percentage of the total incident energy and most of the optical energy goes into the sidebands surrounding the superoscillation spot. One might improve the superoscillation focusing efficiency by using multiple-phase modulation, which can be realized using multiple-step dielectric layers or phase-modulation metasurfaces. Further reducing the superoscillation spot size requires ultra-fine modulation of the light wave front, which is limited by the size of the subwavelength structures for phase modulation. One possible solution is to adopt a planar SOL group instead of a single lens or to employ a specially designed curved-surface refractive lens. The large sidebands seem to be unavoidable if the spot size is much smaller than the superoscillatory criterion, which greatly limits the FOV in superresolution imaging that is based on optical superoscillation. Nevertheless, in the application of superresolution microscopy, the low efficiency is not a practical problem since commercialized photodetectors are sufficiently sensitive for the low optical intensity of the superoscillation fields. The issue of large sideband can also be alleviated by using a confocal configuration in a superresolution microscope system that is based on carefully designed point-spread functions of the illumination and collection lenses.

For the SOL design, especially in the case where the feature size is much smaller than the superoscillation criterion, the precise calculation of the diffraction field is in high demand for SOL with realistic structures. Since fine superoscillatory features result from precise interference of light waves, any discrepancy between the diffraction calculation and the real light propagation might lead to design failure. Moreover, due to the computational consumption, the aperture of SOLs remains limited to several millimetres. The computational complexity become much higher in cases of non-circular symmetry, which involve 2D integrals; a possible solution is to use GPU-based computation and a multi-thread method to accelerate the diffraction calculation. Up to now, most of the reported SOLs are only applicable for a single wavelength. Although SOLs for several isolated wavelengths have been demonstrated either experimentally or theoretically, true broadband achromatic SOLs remain in their infancy. A hybrid lens that integrates both refractive and diffractive lenses may provide a promising paradigm, which will even benefit superoscillation focusing of ultrashort optical pulses. The other challenge for SOLs lies in the small-angle operation; however, wider angle performance might be achieved by carefully designing quasi-continuous phase modulation, which can be used for fast superresolution imaging based on superoscillation. Optical superoscillation provides a new route for realizing superresolution and overcoming the diffraction limit, and it has demonstrated potential in various applications. Further investigation is necessary for understanding the physics behind optical superoscillation phenomena and shedding light on more powerful imaging systems, which may significantly promote the development of optical superoscillatory devices.

## Supplementary information

Reprint Permission 1

Reprint Permission 2

Reprint Permission 3

Reprint Permission 4

Reprint Permission 5

Reprint Permission 6

Reprint Permission 7

Reprint Permission 8

Reprint Permission 9
