# Supplementary material for: Superoscillation: from physics to optical applications
Source: Light Sci Appl. 2019 Jun 12;8:56. doi: 10.1038/s41377-019-0163-9 (PMC6560133; doi:10.1038/s41377-019-0163-9)
Supplement: Supplementary file 4 — Reprint Permission 4 [file 41377_2019_163_MOESM4_ESM.pdf]

Subject:	RE: RE: copyright permission request
From:	pubscopyright <copyright@osa.org> Apr 3, 2019 10:39:00 PM
To:	"晏绍奎" <20180801035@cqu.edu.cn>
Cc:	"eleqc@nus.edu.sg" <eleqc@nus.edu.sg>, "gchen1@cqu.edu.cn" <gchen1@cqu.edu.cn>, "wenzq@cqu.edu.cn" <wenzq@cqu.edu.cn>, pubscopyright <copyright@osa.org>

Dear Cheng-Wei Qiu,

Thank you for the additional information.

For the use of the requested material from **[1]** Gang Chen, Kun Zhang, Anping Yu, Xianyou Wang, Zhihai Zhang, Yuyan Li, Zhongquan Wen, Chen Li, Luru Dai, Senling Jiang, and Feng Lin, "Far-field sub-diffraction focusing lens based on binary amplitude-phase mask for linearly polarized light," Opt. Express24, 11002-11008 (2016), **[2]** Zhixiang Wu, Kun Zhang, Shuo Zhang, Qijian Jin, Zhongquan Wen, Lingfang Wang, Luru Dai, Zhihai Zhang, Hao Chen, Gaofeng Liang, Yufei Liu, and Gang Chen, "Optimization-free approach for generating sub-diffraction quasi-non-diffracting beams," Opt. Express 26, 16585-16599 (2018) and **[3]** Zhixiang Wu, Qijian Jin, Shuo Zhang, Kun Zhang, Lingfang Wang, Luru Dai, Zhongquan Wen, Zhihai Zhang, Gaofeng Liang, Yufei Liu, and Gang Chen, "Generating a three-dimensional hollow spot with sub-diffraction transverse size by a focused cylindrical vector wave," Opt. Express 26, 7866-7875 (2018):

> Because you, or your coauthors Gang Chen and Zhongquan Wen, are the author of the source paper from which you wish to reproduce material, OSA considers your requested use of its copyrighted materials to be permissible within the author rights granted in the Copyright Transfer Agreement submitted by the requester on acceptance for publication of his/her manuscript. It is requested that a complete citation of the original material be included in any publication. This permission assumes that the material was not reproduced from another source when published in the original publication.

For the use of figures 6 and 9 from **[4]** Xiao Han Dong, Alex M. H. Wong, Minseok Kim, and George V. Eleftheriades, "Superresolution far-field imaging of complex objects using reduced superoscillating ripples," Optica 4, 1126-1133 (2017):

> OSA considers your requested use of its copyrighted material to be Fair Use under United States Copyright Law. It is requested that a complete citation of the original material be included in any publication.

While your publisher should be able to provide additional guidance, OSA prefers the below citation formats:

> For citations in figure captions:
>
> [Reprinted/Adapted] with permission from ref [x], [Publisher]. (with full citation in reference list)
>
> For images without captions:
>
> Journal Vol. #, first page (year published) An example: Optica 4, 1126 (2017)

Please let me know if you have any questions.

Kind Regards,

Rebecca Robinson

Rebecca Robinson

April 3, 2019

Authorized Agent, The Optical Society

The Optical Society (OSA)

2010 Massachusetts Ave., NW

Washington, DC 20036 USA

www.osa.org

Reflecting a Century of Innovation

The Optical Society | Since 1916

From: 晏绍奎 <20180801035@cqu.edu.cn>
Sent: Wednesday, April 3, 2019 10:31 AM
To: pubscopyright <copyright@osa.org>
Subject: Re: RE: copyright permission request

Dear Sir,

I apologize for not given you the full information, now I will giving you the whole information of the requestor

The requestor name: Gang Chen[1], Zhongquan Wen[1] and Cheng-Wei Qiu[2*]

[1]College of Optoelectronics Engineering, Chongqing University, 174 Shangzheng Street, Chongqing 400044, China gchen1@cqu.edu.cn, wenzq@cqu.edu.cn

[2]Department of Electrical and Computer Engineering, National University of Singapore, 4 Engineering Drive 3, Singapore 117583, Singapore

Correspondence: Cheng-Wei Qiu(eleqc@nus.edu.sg)

If any other information is required, You can contact me by sending me a mail all the requested details to 20180801035@cqu.edu.cn.

I am Looking forward to your reply.

Thank you.

Yours Sincerely.

-----原始邮件-----
发件人:pubscopyright <copyright@osa.org>
发送时间:2019-04-03 02:43:19 (星期三)
收件人: "晏绍奎" <20180801035@cqu.edu.cn>, pubscopyright <copyright@osa.org>
抄送:
主题: RE: copyright permission request

Thank you for contacting The Optical Society.

Unfortunately, your signature block was not transmitted with your request. To help OSA assess your request, could you provide the name of the requestors?

Thank you,

Rebecca Robinson

Rebecca Robinson

April 2, 2019

Authorized Agent, The Optical Society

The Optical Society (OSA)

2010 Massachusetts Ave., NW

Washington, DC 20036 USA

www.osa.org

Reflecting a Century of Innovation

OSA The Optical Society | 100 Since 1916

From: 晏绍奎 <20180801035@cqu.edu.cn>
Sent: Thursday, March 21, 2019 11:00 AM
To: pubscopyright <copyright@osa.org>
Subject: copyright permission request

Dear Sir,

I am writing to inquire about the copyright permission request,We have quoted some pictures from your journal articles for my paper to be published,we will provide the title of the paper and the figure number citated for copyright transform.

1、Title：Far-field sub-diffraction focusing lens based on binary amplitude-phase mask for linearly polarized light.

Figure：Figure. 4 ,Figure. 5,Figure. 6

2、Title：Optimization-free approach for generating sub-diffraction quasi-non-diffracting beams.

Figure：Figure. 2,Figure. 6, Figure. 8, Figure. 9, Figure. 10

3、Title：Generating a three-dimensional hollow spot with sub-diffraction transverse size by a focused cylindrical vector wave.

Figure：Figure. 3,Figure. 5,Figure. 6

4、Title: Superresolution far-field imaging of complex objects using reduced superoscillating ripples.

Figure: Figure. 6,Figure. 9

If any other information is required, You can contact me by sending me a mail all the requested details to20180801035@cqu.edu.cn.

The information of author：*College of Optoelectronics Engineering, Chongqing University, 174 Shazheng Street, Chongqing 400044, China*

gchen1@cqu.edu.cn

I am Looking forward to your reply.

Thank you.

Yours Sincerely.
