# Supplementary material for: Superoscillation: from physics to optical applications
Source: Light Sci Appl. 2019 Jun 12;8:56. doi: 10.1038/s41377-019-0163-9 (PMC6560133; doi:10.1038/s41377-019-0163-9)
Supplement: Supplementary file 9 — Reprint Permission 9 [file 41377_2019_163_MOESM9_ESM.pdf]

| | |
|---|---|
| 主　题: | Re: Request for reprint permission of figures in Scientific Reports |
| 发件人: | "Creative Commons" <info@creativecommons.org> 2019-4-23 22:17:15 |
| 收件人: | gchen1@cqu.edu.cn |

Hey 陈刚,

When you find a CC licensed work that you wish to reuse, simply read the license and comply with those terms. We have more than one license so be sure to do your due diligence to find out which one is on the work you wish to reuse.

For instance, say you wanted to use this image of my cat. You can view the license by clicking on "Some Rights Reserved" -- you can read there that all you must do is give me credit and you have permission to reuse the image.

Here are some tips for giving credit: https://wiki.creativecommons.org/wiki/Best_practices_for_attribution

Hope this helps!

Mari

---
Creative Commons

On Tue, 23 Apr at 1:59 AM , 陈刚 <gchen1@cqu.edu.cn> wrote:
Dear Sir/Madam,
I am writing to request for the reprint permission of reusing some figures published in Scientific Reports. We have one review article to be published in Light: science & applications. In the review article, we reused some of the figures published in Scientific Reports as listed below.
We also sending you the information of the authors' information of the review article

The requesters' names: Gang Chen[1], Zhongquan Wen[1] and Cheng-Wei Qiu[2*]
[1]College of Optoelectronics Engineering, Chongqing University, 174 Shangzheng Street, Chongqing 400044, China
gchen1@cqu.edu.cn,wenzq@cqu.edu.cn
[2]Department of Electrical and Computer Engineering, National University of Singapore, 4 Engineering Drive 3, Singapore 117583, Singapore
Correspondence: Cheng-Wei Qiu(eleqc@nus.edu.sg)

Could you please send us the reprint permissions of following figures as soon as possible, since we only have 9 days before the deadline of revision submission.
Thanks a lot!

1、Title:Super-oscillatory focusing of circularly polarized light by ultralong focal length planar lens based on binary amplitude-phase modulation, *Scientific Reports* 6, 29068 (2016)
Figures: Fig.3, Fig.4a, Fig.4b and Fig.4c
2、Title:Generation of a sub-diffraction hollow ring by shaping an azimuthally polarized wave, *Scientific Reports* 6, 37776(2016)
Figures: Fig.4a, Fig.6b and Fig.6c
3.Title:Creation of Sub-diffraction Longitudinally Polarized Spot by Focusing Radially Polarized Light with Binary Phase Lens, *Scientific Reports* 6,38859(2016)
Figures: Fig.5a, Fig.6a and Fig.6b
4:Title Super-resolution optical telescopes with local light diffraction shrinkage, *Scientific Reports* 5, 18485(2015)
Figures: Fig.1a, Fig.4a, Fig.4b and Fig.4c

If further information is required, please let me know.
I am Looking forward to your reply.
Regards!
Sincerely

**Gang Chen, PhD, Professor (PhD Advisor), Director**
Department of Optoelectronic Information Engineering,
School of Optoelectronic Engineering, Chongqing University (Campus A).
Key Laboratory of Optoelectronic Technology & System (Ministry of Education, China).
Street174 Shazheng Street
Shapingba, Chongqing 400044
China
Tele (Work)：+862365111022, Fax (Work)+862365104131
Cell :+8618702323159
email：GCHEN1@cqu.edu.cn
web1：http://coe.cqu.edu.cn/gdmainweb/shownews.aspx?siteId=1&L1name=Teacher&L1Id=20161118022018VQJ84E&kind=c&L2name=Teacher&L2Id=20161118022103QRUIMR&infoId=20161122094352ZSVHXQ
web2：http://yz.cqu.edu.cn/view_teacher.php?tid=08032

This page is available in the following languages:

# Creative Commons License D[illegible]

## Attribution 4.0 International (CC BY 4.0)

This is a human-readable summary of (and not a substitute for) the license.

## You are free to:

**Share** — copy and redistribute the material in any medium or format

**Adapt** — remix, transform, and build upon the material

for any purpose, even commercially.

The licensor cannot revoke these freedoms as long as you follow the license terms.

## Under the following terms:

**Attribution** — You must give appropriate credit, provide a link to the license, and indicate if changes were made. You may do so in any reasonable manner, but not in any way that suggests the licensor endorses you or your use.

**No additional restrictions** — You may not apply legal terms or technological measures that legally restrict others from doing anything the license permits.

## Notices:

You do not have to comply with the license for elements of the material in the public domain or where your use is permitted by an applicable exception or limitation.

No warranties are given. The license may not give you all of the permissions necessary for your intended use. For example, other rights such as publicity, privacy, or moral rights may limit how you use the material.
